# Factors Predictive of Early Discontinuation of Preventive Treatment in Children With Household Exposure to Multidrug-resistant Tuberculosis

**DOI:** 10.1093/ofid/ofaf425

**Published:** 2025-07-18

**Authors:** Trinh Duong, Joanna Brigden, Susan E Purchase, Neil A Martinson, Lee Fairlie, Suzanne Staples, Faeezah Patel, Nadia Sabet, Charlotte Layton, Thomas Wilkinson, H Simon Schaaf, James A Seddon, Anneke C Hesseling

**Affiliations:** Institute of Clinical Trials and Methodology, Faculty of Population Health Sciences, University College London, London, UK; Institute of Clinical Trials and Methodology, Faculty of Population Health Sciences, University College London, London, UK; Desmond Tutu TB Centre, Department of Paediatrics and Child Health, Stellenbosch University, Cape Town, South Africa; Perinatal HIV Research Unit, University of the Witwatersrand, Johannesburg, South Africa; Center for Tuberculosis Research, Department of Medicine, Johns Hopkins University School of Medicine, Baltimore, Maryland, USA; Wits RHI, Faculty of Health Sciences, University of the Witwatersrand, Johannesburg, South Africa; Tuberculosis & HIV Investigative Network, Research Department, Durban, South Africa; Wits RHI, Faculty of Health Sciences, University of the Witwatersrand, Johannesburg, South Africa; Perinatal HIV Research Unit, University of the Witwatersrand, Johannesburg, South Africa; Institute of Clinical Trials and Methodology, Faculty of Population Health Sciences, University College London, London, UK; Health Economics Unit, University of Cape Town, Cape Town, South Africa; Desmond Tutu TB Centre, Department of Paediatrics and Child Health, Stellenbosch University, Cape Town, South Africa; Desmond Tutu TB Centre, Department of Paediatrics and Child Health, Stellenbosch University, Cape Town, South Africa; Department of Infectious Disease, Imperial College London, London, UK; Desmond Tutu TB Centre, Department of Paediatrics and Child Health, Stellenbosch University, Cape Town, South Africa

**Keywords:** adherence, children, levofloxacin, multidrug-resistant tuberculosis, preventive treatment

## Abstract

**Background:**

The World Health Organization recommended levofloxacin for tuberculosis (TB) preventive treatment for child and adult contacts of multidrug-resistant TB.

**Method:**

TB-CHAMP (ISRCTN92634082) was a double-blind community-based multisite randomized placebo-controlled trial assessing levofloxacin as preventive treatment in children with household exposure to adults with microbiologically confirmed multidrug-resistant TB in South Africa. Households were randomized 1:1 to 24 weeks of daily levofloxacin (adult scored 250-mg tablets) versus placebo. Treatment adherence was ascertained through pill counts and treatment cards. Competing risk methods were used to assess factors associated with early treatment discontinuation for nonclinical reasons before achieving ≥80% of allocated doses (adequate treatment).

**Results:**

Among 911 of 922 children included in analysis, 90% were younger than 5 years of age. Overall, 765 (84%) of children achieved adequate treatment, 135 (15%) discontinued treatment early, and 11 (1%) had not achieved adequate treatment by the end-of-treatment period. Sixty-four (7%) children stopped for clinical reasons and 71 (8%) for nonclinical reasons, with similar proportions across treatment groups. Baseline factors associated with early treatment discontinuation for nonclinical reasons were previous receipt of herbal/traditional medicine (subhazard ratio 3.08; 95% confidence interval, 1.69–5.59; *P* < .001), and caregivers reporting difficulties administering medication (subhazard ratio 2.73; 1.11–6.71; *P* = .029). Children with poor treatment adherence by week 4 were more likely to subsequently stop treatment early for nonclinical reasons (subhazard ratio 2.72; 1.06–6.97; *P* = .037).

**Conclusions:**

Adherence to the 250-mg levofloxacin formulation was good among young children on preventive TB therapy. Adherence support for children and caregivers, and addressing early signs of poor adherence, may enhance treatment completion.

## BACKGROUND

Tuberculosis (TB) is the leading cause of mortality from a single infectious agent worldwide [1]. Children, particularly young children aged younger than 5 years, are at higher risk of progression to TB after exposure to *Mycobacterium tuberculosis* (*Mtb*) compared to adults and are more prone to severe forms of disease [2, 3]. Each year, an estimated 30 000 children develop multidrug-resistant (MDR)-TB, defined as disease caused by *Mtb* resistant to isoniazid and rifampicin [4]. MDR-TB is a major threat to global TB control and is more complex and expensive to treat than drug-susceptible TB [5]. Following evidence from randomized trials, the World Health Organization strongly recommended TB preventive treatment (TPT) with 6-month daily levofloxacin for individuals exposed to people with rifampicin-resistant or MDR-TB [6–9].

Noncompletion of TPT is associated with poorer clinical outcomes [10, 11]. A post hoc analysis of 2 TPT randomized trials showed TB incidence was nearly 3-fold higher among participants not completing treatment compared to those who did. This may be particularly pertinent for levofloxacin preventive treatment, given the relatively long treatment duration. Studies under programmatic settings showed completion rates of drug-susceptible TPT among children were generally poor for isoniazid monotherapy, usually given for 6 months, but better for shorter 3- to 4-month regimens [12–14].

Identifying predictors of MDR-TPT noncompletion among children may inform efforts to enhance treatment adherence. Furthermore, treatment acceptability for the child and caregivers play an important role in treatment adherence, with key barriers to adherence including pill size and bitter taste, long duration of therapy, and perceived medication side effects [12, 15–18]. Although the pediatric dispersible formulation of levofloxacin is available, the adult solid formulations remain widely used in children globally because of affordability but are less palatable [10, 19].

In the randomized Tuberculosis Child Multidrug-Resistant Preventive Therapy Trial (TB-CHAMP; ISRCTN92634082) enrolling child household MDR-TB contacts, participants received either the 250-mg adult levofloxacin formulation or matching placebo [9]. A nested qualitative study within the trial previously found the adult formulation was disliked by many younger children because of the bitter taste but was acceptable to those who could swallow the tablet whole [17]. The main aim of this quantitative analysis was to assess factors associated with treatment noncompletion and poor adherence in the overall study cohort.

## METHODS

### Study Design

TB-CHAMP was a phase 3 double-blind randomized controlled trial enrolling children with household exposure to adult MDR-TB patients across 5 sites in South Africa. The study design has been previously described and the results reported [9, 20].

Initially, only children aged younger than 5 years with household exposure to an adult with microbiologically confirmed pulmonary MDR-TB were eligible, regardless of *Mtb* infection status. Later, older children and adolescents aged 5–17 years who either tested positive on interferon-gamma release assay (QuantiFERON-TB Gold Plus, Qiagen) or were living with HIV were included.

Participants were randomized at household level in 1:1 ratio to levofloxacin or placebo, stratified by site. Study medications were administered daily for 24 weeks, as the 250-mg scored tablets for levofloxacin or matching placebo, both manufactured by Macleods Pharmaceuticals, India. Dosing was based on 15–20 mg/kg (maximum 750 mg) daily using predefined weight bands (Supplementary Table 1). The initial dosing was given at the study site under observation of study staff, and subsequent doses by caregivers at home. Adequate treatment was defined as taking at least 80% of allocated study treatment doses (≥134/168 doses). Treatment interruptions of up to 4 weeks overall were allowed, resulting in 28-week maximum treatment duration.

Follow-up visits were at 4, 8, 12, 16, 24, 36, 48, and 72 weeks. Adherence was verified through pill counts and reported adherence based on treatment cards at each study visit. Sites’ staff estimated the number of days that study treatment was not taken from this information. Adherence support was provided at every study visit and between visits as required. If there were any concerns (eg, too many tablets returned), the caregivers were counseled, with the reason for poor adherence sought and potential solutions put in place. Households that were struggling with adherence were phoned and seen more frequently. Participants may stop treatment early for clinical reasons (including late screening failure, presumed or diagnosed TB disease, adverse events, diagnosis of an infectious adult with drug-susceptible TB in the same household), or for nonclinical reasons (including withdrawal of consent to treatment by the child's parents or legal guardians, withdrawal of assent for treatment by the child, relocation, and inadequate adherence to the protocol treatment).

Treatment acceptability among the children and their caregivers was assessed using a questionnaire administered at baseline and selected time points during the treatment period [21]. Responses were recorded as ranked categories, for example, how the child appears to feel about the taste of medication reported as “dislike very much,” “dislike,” “neutral,” “like,” or “like very much.”

### Statistical Analysis

Participants were assigned to 1 of the following treatment completion outcomes: achieved adequate treatment (ie, took ≥80% of the 168 doses); early discontinuation of treatment for a nonclinical reason (eg, loss to follow-up) before achieving adequate treatment; early discontinuation of treatment for a clinical reason (eg, development of TB disease) before achieving adequate treatment; and reached end-of-treatment phase, but with <80% of doses taken.

Adherence while on treatment was defined as the proportion of doses taken compared to the number of doses prescribed while the participant was on treatment; for example, if a participant stopped treatment at week 6 after developing TB having previously missed 2 doses, their adherence while on treatment would be 95% (Supplementary Figure 1). This was then categorized as ≥90%, 80 to <90%, and <80%.

Separate analyses were undertaken using competing risk methods to estimate, by study treatment groups, the cumulative incidence of early discontinuation of treatment for clinical and for nonclinical reasons before achieving adequate treatment. Competing risk methods take into account, for example, that a participant who stopped treatment early for clinical reason(s) can no longer discontinue treatment for nonclinical reason(s). Standard Kaplan-Meier survival analysis overestimates the cumulative incidence in the presence of competing events [22, 23]. The difference between treatment groups was assessed using Fine and Gray models [24, 25], adjusting for site (stratification factor) and age, with household clustering accounted for. Participants were analyzed according to the study drug prescribed.

Potential baseline predictors of early discontinuation of treatment for nonclinical reasons were assessed first. Separate analyses then assessed the association between potential factors at the week 4 visit and subsequent early discontinuation of treatment for nonclinical reasons. Here, only children who attended the week 4 visit and were still on treatment at the time were included, with time zero set to the week 4 visit date. Ordinal logistic regression was used to compare adherence while on treatment between treatment groups and to assess factors at baseline associated with adherence.

Selected factors at baseline, chosen on the basis of plausibility and previous studies [21, 26], were assessed and included: demographic characteristics of the child participant, clinical factors (eg, comorbidities, hospitalization), previous use of herbal/traditional medicine, factors relating to exposure to the index patient (eg, whether slept in the same bed or room), HIV infection status of mother, socioeconomic status (SES) index of the household, and treatment acceptability. A SES index, a continuous score, was defined for each TB-CHAMP household. This was derived by applying the principle component analysis coefficients of questions in the South African Demographic and Health Survey 2016 to applicable characteristics of each TB-CHAMP household, such as house type/material, access to water, basic household assets, and type of toilet facilities [27]. The TB-CHAMP household SES index was then translated to quintiles of South African Demographic and Health Survey households to allow comparison of the SES of TB-CHAMP households to the general South African population, with the first quintile representing the lowest SES score. Acceptability responses were analyzed as binary variables, by combining where relevant, the 2 most negative categories (eg, “dislike very much” and “dislike”) versus the rest.

Potential predictors assessed at the week-4 visit included treatment acceptability, behavioral factors, and whether the participant experienced any adverse events after randomization. The behavioral factors considered were poor adherence (defined as taking <80% of prescribed doses by the week 4 visit), not bringing the pill bottle as instructed, and not bringing the treatment card.

All factors with *P* < .2 in univariable analysis were assessed in multivariable models [28]. Treatment group was adjusted for, along with a priori confounders, which were site and age group (predefined as <3, 3 to <5, ≥5 years). Treatment acceptability was not adjusted for when assessing the potential effect of other factors since it could be on the causal pathway.

Participants who received their last dose at least 134 days after the first dose but were missing information on the number of doses taken were excluded from all analyses since their treatment completion outcome could not be ascertained. For a small number of children with missing information on the number of doses taken but took their last dose within 133 days of the first dose (ie, < 80% of overall 168 doses), it was assumed treatment was discontinued early before achieving adequate treatment; these children were included in the analyses of early treatment discontinuation, but not that of adherence while on treatment. Multiple testing was not accounted for. Analyses were performed with Stata version 18.0 (StataCorp, College Station, TX).

### Patient Consent Statement

The trial was approved by the Health Research Ethics Committee of Stellenbosch University (M16/02/009) and the University of the Witwatersrand (160409), the South African Health Products Regulatory Agency (20160128), and the South African Department of Health (DOH-27-0117-5309). Informed written consent was provided by all index patients and participants’ parents or legal guardians, and assent was also obtained from children ≥7 years.

## RESULTS

A total of 922 children from 497 households were enrolled into TB-CHAMP. The analyses presented here included 911 (99%) children with the treatment completion outcome known; Supplementary Figure 2. One participant who was randomly assigned to the levofloxacin group but prescribed placebo in error was included under the placebo group for the purpose of these analyses. Eleven children for whom treatment completion outcome could not be ascertained were excluded.

The baseline characteristics were similar between treatment groups (Table 1 and Supplementary Table 2). Overall, the median age was 2.8 years (interquartile range 1.4–4.2 years), with 90% of children younger than 5 years of age. Fifty-one percent of children were female. HIV prevalence was low at 2%, though more than one third of the cohort with relevant information available on maternal HIV status were HIV exposed but uninfected.

**Table 1.** Characteristics of Participants at Baseline (N = 911^a^) [see Author's comment #3 in separate email]

		Levofloxacin	Placebo	Total
Number of children	N	448 (100%)	463 (100%)^b^	911 (100%)
Sex	Male	211 (47%)	236 (51%)	447 (49%)
	Female	237 (53%)	227 (49%)	464 (51%)
Age (y)	Median (IQR)	3.0 (1.4,4.3)	2.6 (1.3, 4.1)	2.8 (1.4, 4.2)
	<1	82 (18%)	83 (18%)	165 (18%)
	1 to <3	140 (31%)	173 (37%)	313 (34%)
	3 to <5	179 (40%)	172 (37%)	351 (39%)
	5 to <10	18 (4%)	17 (4%)	35 (4%)
	10 to <18	29 (6%)	18 (4%)	47 (5%)
IGRA result^c^	Negative	294 (67%)	331 (74%)	625 (70%)
	Positive	133 (30%)	106 (24%)	239 (27%)
	Indeterminate	11 (3%)	12 (3%)	23 (3%)
	Missing data	10	14	24
Mother's HIV status	Negative	250 (61%)	277 (62%)	527 (62%)
	Positive	162 (39%)	167 (38%)	329 (38%)
	Missing data	36	19	55
Co-morbidities	No	425 (95%)	433 (94%)	858 (94%)
	HIV-positive	10 (2%)	9 (2%)	19 (2%)
	Other	13 (3%)	21 (5%)	34 (4%)
Previously received TB treatment	No	438 (98%)	455 (98%)	893 (98%)
	Yes	10 (2%)	8 (2%)	18 (2%)
Previously received herbal or traditional medicine	No	390 (87%)	402 (87%)	792 (87%)
	Yes	58 (13%)	60 (13%)	118 (13%)
	Missing data	0	1	1
Relationship of index patient to child participant	Mother	78 (17%)	81 (17%)	159 (17%)
	Father	49 (11%)	43 (9%)	92 (10%)
	Other family member	273 (61%)	286 (62%)	559 (61%)
	Other nonfamily member	48 (11%)	53 (11%)	101 (11%)
Whether index patient is the primary caregiver	Primary carer	100 (22%)	101 (22%)	201 (22%)
	Not primary carer, regularly cares for child	189 (42%)	215 (46%)	404 (44%)
	Neither	158 (35%)	147 (32%)	305 (34%)
	Missing data	1	0	1
Slept in the same bed or same room as index patient	Not same room	259 (58%)	303 (65%)	562 (62%)
	Same room, not same bed	61 (14%)	36 (8%)	97 (11%)
	Slept same bed	128 (29%)	124 (27%)	252 (28%)
Coughing history of index patient	No current cough	224 (50%)	224 (49%)	448 (50%)
	Current cough <4 wk	87 (20%)	119 (26%)	206 (23%)
	Current cough ≥4 wk	135 (30%)	116 (25%)	251 (28%)
	Missing data	2	4	6
Socioeconomic status quintile^d^	1st	27 (6%)	23 (5%)	50 (6%)
	2nd	96 (21%)	95 (21%)	191 (21%)
	3rd	221 (49%)	227 (49%)	448 (49%)
	4th	104 (23%)	116 (25%)	220 (24%)
	5th	0 (0%)	0 (0%)	0 (0%)
	Missing data	0	2	2
Site	DTTC	221 (49%)	229 (49%)	450 (49%)
	Shandukani	90 (20%)	76 (16%)	166 (18%)
	Matlosana	115 (26%)	141 (30%)	256 (28%)
	ILTBRU	3 (1%)	4 (1%)	7 (1%)
	THINK	19 (4%)	13 (3%)	32 (4%)

Percentages were based on participants with information available.

Abbreviations: DTTC, Desmond Tutu TB Centre; ILTBRU, Isanga Lethemba TB Research Unit; IQR, interquartile range; THINK, TB and HIV Investigative Network.

^a^Analyses excluded 11/922 children in whom treatment completion outcome could not be ascertained.

^b^One participant was randomly assigned to levofloxacin group but prescribed placebo in error, and for the purpose of these analyses, was analyzed under the placebo group.

^c^ *Mycobacterium tuberculosis* infection status at baseline was assessed with the use of the QuantiFERON-Gold Plus (Qiagen) interferon-γ release assay (IGRA).

^d^The TB-CHAMP household SES index score was categorized according to the quintiles of households in the South African Demographic and Health Survey 2016 survey, with the first quintile representing the lowest SES score.

Previous use of herbal/traditional medicine was reported in 13% of children, though this proportion varied by sites (being greater in more rural areas than urban areas), and was likely to be underreported among older children (Supplementary Table 3). The most reported reasons for herbal/traditional medicine use were “prophylaxis” and alleviating wind in infants (Supplementary Table 4). Fifteen percent of children received antibiotics in the 2 weeks before enrolment (Supplementary Table 2).

For just over a quarter of children, the MDR-TB index patient was either their mother (17%) or father (10%). In most participants, the index patient was the primary caregiver (22%) or otherwise regularly cared for the child (44%). Approximately 40% of children slept in the same room as the index patient.

**Figure 1.**
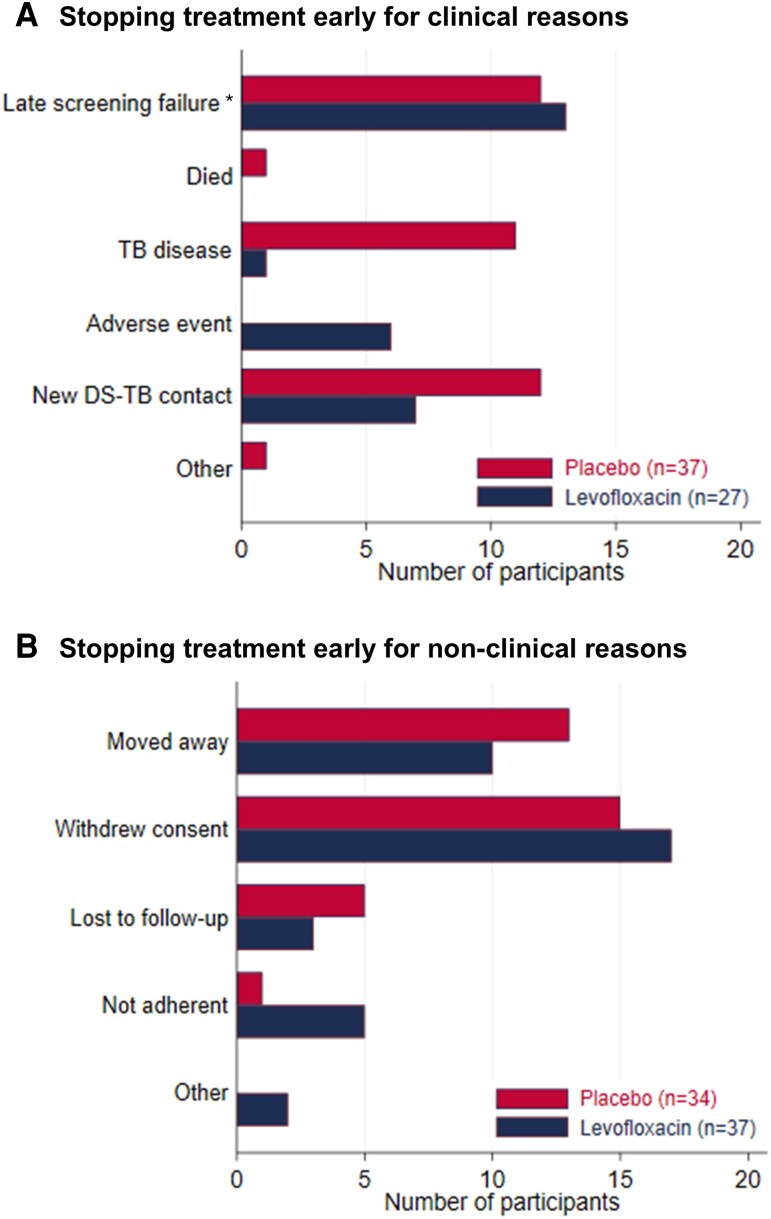
Reasons for early discontinuation of treatment before achieving adequate treatment: A) Clinical reasons B) Non-clinical reasons. *14 of the 25 participants who were late screening failures were because of a delay in isoniazid susceptibility test result for the index patient (see Supplementary Table 5). Abbreviation: DS-TB, drug-susceptible tuberculosis.

Around 85% (765/911) of children achieved adequate treatment (≥80% of the 168 doses), with similar proportions in the levofloxacin group (377/448, 84.2%) and placebo group (388/463, 83.8%); Table 2. Overall, 135 (15%) children discontinued treatment early before achieving adequate treatment, whereas 11 (1%) reached end-of-treatment phase (ie, 28 weeks) without achieving adequate treatment. Sixty-four (7%) children stopped treatment early for clinical reasons, including 12 because of presumed/diagnosed TB disease after randomization and 6 (in the levofloxacin group) for adverse events (Figure 1, Supplementary Table 5). Seventy-one (7.8%) stopped for nonclinical reasons, of whom 32 (45%) discontinued following withdrawal of consent, 23 (32%) because of moving away and 8 (11%) due to loss to follow-up.

**Table 2.** Treatment Completion Outcomes

	Levofloxacin	Placebo	Overall
Children randomized	452	470	922
Children with treatment completion outcome ascertained	448	463	911
Achieved ≥80% of overall allocated doses	377 (84.2%)	388 (83.8%)	765 (84.0%)
Discontinued treatment early for clinical reasons^a^	27 (6.0%)	37 (8.0%)	64 (7.0%)
Discontinued treatment early for non-clinical reasons^a^	37 (8.3%)	34 (7.3%)	71 (7.8%)
Reached end of treatment phase, but took <80% allocated doses	7 (1.6%)	4 (0.9%)	11 (1.2%)^b^

^a^Before reaching 80% of overall 168 allocated doses. The reasons for stopping treatment early are summarized in Figure 1.

^b^All took ≥50% of doses, 9 of 11 took ≥70%.

**Figure 2.**
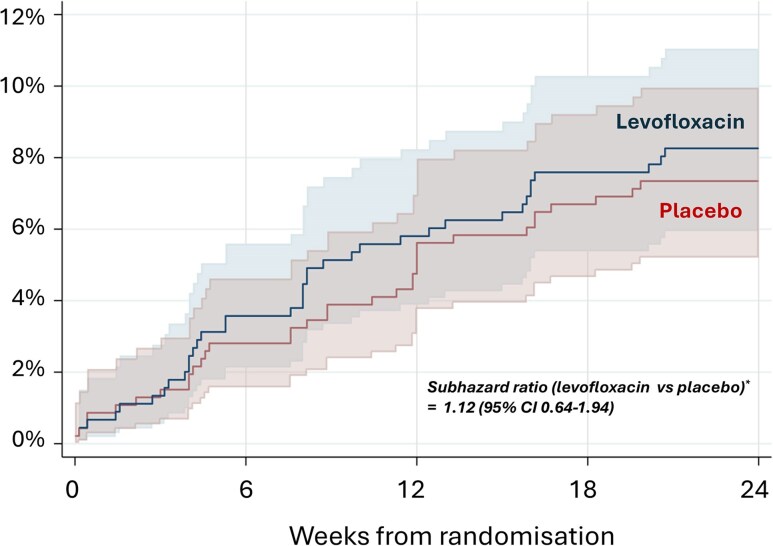
Cumulative incidence of early discontinuation of treatment for nonclinical reason(s) before achieving adequate treatment (with 95% confidence bands). *The subhazard ratio was derived from Fine-Gray model and corresponds to the relative difference in the instantaneous rate of early discontinuation of treatment for nonclinical reason(s) among participants who have not yet stopped treatment early for nonclinical reason(s) at a given time point [25]. Please refer to Supplementary Figure 3 in the Supplement for the cumulative incidence of early discontinuation of treatment for clinical reason(s). Abbreviation: CI, confidence interval.

The likelihood of stopping treatment early for nonclinical reasons before achieving adequate treatment was similar between treatment groups, subhazard ratio comparing levofloxacin versus placebo 1.12 (95% confidence interval [CI], .64–1.94); Figure 2. Overall, the cumulative incidence of stopping treatment early for a nonclinical reason was 5.7% (95% CI, 4.3–7.3) by 12 weeks across both treatment groups combined, and 7.8% (6.2%–9.7%) by 24 weeks.

In multivariable analyses, children who had ever received herbal/traditional medicine at baseline were more likely to stop treatment early for nonclinical reasons, subhazard ratio 3.08 (95% CI, 1.69–5.59; *P* < .001; Table 3). This association remained in separate analyses restricted to: (1) children aged <2 years (subhazard ratio 4.48; 95% CI, 2.11–9.52; *P* < .001) and (2) participants from the study site (Matlosana) with highest prevalence of herbal/traditional medicine use (subhazard ratio 5.20; 1.17–23.03; *P* = .03). There was weak evidence that early discontinuation of treatment for nonclinical reasons was less likely to occur among children aged 5 years or older (*P* = .105; Table 3), those whose mothers were living with HIV (*P* = .073), and in children exposed to TB index patients with a current cough at baseline (*P* = .082). Differences between sites remained after adjustment for other factors.

**Table 3.** Multivariable Analyses of Factors Associated With Early Discontinuation of Treatment for Nonclinical Reasons [see Author's comment #5]

Factors^a^		Number of Children	Discontinued Treatment Early for Nonclinical Reasons (%)	Subhazard Ratio (95% Confidence Interval)^b^	*P*
Overall		911	71 (8%)	**…**	
1. Factors at baseline					
a. Demographic, clinical, and TB exposure					
Age (y)	<3 y	478	40 (8%)	1	.105^c^
	3 to <5	351	27 (8%)	1.02 (0.61–1.69)	…
	≥5 y	82	4 (5%)	0.31 (0.10–0.95)	…
Mother's HIV status	Negative	527	47 (9%)	1	.073
	Positive	329	19 (6%)	0.59 (0.33–1.05)	…
Ever received herbal or traditional medicine	No	792	56 (7%)	1	<.001
	Yes	118	15 (13%)	3.08 (1.69–5.59)	…
Coughing history of index patient	No current cough	448	46 (10%)	1	.082
	Current cough <4 wk	206	11 (5%)	0.56 (0.25–1.25)	…
	Current cough ≥4 wk	251	12 (5%)	0.48 (0.23–1.01)	…
Site^d^	DTTC	450	40 (9%)	1	<.001
	Shandukani	166	17 (10%)	1.09 (0.57–2.10)	…
	Matlosana	256	9 (4%)	0.27 (0.10–0.76)	…
	THINK	32	5 (16%)	3.51 (1.25–9.87)	…
b. Acceptability					
Caregiver found it very difficult or difficult to administer dose	No	768	60 (8%)	1	.029
	Yes	29	6 (21%)	2.73 (1.11–6.71)	…
Child had to be coerced or forced to take medication	No	632	46 (7%)	1	.052
	Yes	164	19 (12%)	1.91 (0.99–3.66)	…
Caregiver felt the size of the tablet was too big or a bit big	No	782	63 (5%)	1	.075
	Yes	15	3 (20%)	3.27 (0.89–12.03)	…
2. Factors at wk 4 visit^e^					
a. Behavioral factors		…	…	…	
Poor adherence by wk 4 visit^f^	No	723	27 (4%)	1	.037
	Yes	118	12 (10%)	2.72 (1.06–6.97)	…
Treatment card not returned	No	829	35 (4%)	1	.006
	Yes	21	5 (24%)	7.47 (1.80–30.92)	…
Not bringing pill bottle	No	823	37 (4%)	1	.029
	Yes	27	3 (11%)	3.75 (1.14–12.31)	…
b. Acceptability					
Caregiver found it very difficult or difficult to prepare of study medication	No	824	33 (4%)	1	.063
	Yes	14	3 (21%)	3.54 (0.93–13.42)	…
Caregiver felt the size of the tablet was too big or a bit big	No	825	34 (4%)	1	.022
	Yes	13	2 (15%)	4.82 (1.26–18.45)	…

^a^Factors with *P* < .2 in multivariable analysis and a priori confounders (age group and site) are presented.

^b^Derived from Fine-Gray regression model.

^c^In a post hoc analysis comparing age ≥5 versus <5 y, the subhazard ratio was 0.31 (95% CI, .10–.92), *P* = .03.

^d^The analysis comparing sites excluded 1 site (ILTBRU) with 7 children, none of whom stopped treatment early for nonclinical reasons.

^e^Analyses excluded 61 children who either had stopped treatment before wk 4 visit (n = 19), or did not attend the wk 4 visit (n = 42); see Supplementary Figure 2.

^f^Defined as taking <80% of doses prescribed by the wk 4 visit; see Supplementary Figure 1.

After the first study drug dose was provided at the baseline visit, a small proportion of children (29/797 [4%] with information available) had caregiver-reported difficulties with administering the study medication. These children were more likely to subsequently stop treatment early, subhazard ratio 2.73 (95% CI, 1.11–6.71; *P* = .029; Table 3). There was some evidence that children who had to be coerced or forced to take the medication were also more likely to stop treatment early, subhazard ratio 1.91 (.99–3.66; *P* = .052).

A total of 850 children attended the week 4 visit and were still on study treatment at the time (Supplementary Figure 2). Among these, children who had poor adherence by the week 4 visit were more likely to subsequently stop treatment early for nonclinical reasons, subhazard ratio 2.72 (1.06–6.97, *P* = .037); Table 3. Not returning the treatment card and not returning the pill bottles at the week 4 visit were both also associated with stopping treatment early for nonclinical reasons. At week 4, a small number of children had caregivers reporting difficulties preparing the medication or who found the size of the tablet was big; these children were more likely to subsequently stop treatment early for nonclinical reasons, though data were sparse.

The analysis of adherence while on treatment excluded 1 child who did not start study treatment and another 23 children missing information on the number of doses taken (Supplementary Figure 2). While children were on treatment, their adherence was excellent; overall, the proportion of children taking ≥90% of prescribed doses, 80 to <80%, and <80% were 85.3%, 10.5%, and 4.2%, respectively. Compared to the placebo group, children receiving levofloxacin had poorer adherence while on treatment, odds ratio 1.60 (95% CI, 1.04–2.45); Supplementary Table 6. There were also differences between study sites, but otherwise no other baseline factors were found to be associated with adherence while on treatment (Supplementary Table 7).

## DISCUSSION

Among children receiving 6-month daily MDR-TB preventive treatment with the levofloxacin adult formulation within the TB-CHAMP trial, approximately 85% achieved adequate treatment, in line with the target product profile for TPT recommended by the World Health Organization [29]. Less than 10% discontinued treatment early for nonclinical reasons and very few stopped for adverse events. While the children were still on treatment, adherence was excellent with nearly all taking at least 80% of the doses prescribed during that time.

The treatment completion rate in TB-CHAMP was similar to that among children receiving 9 months of isoniazid as drug-susceptible TPT in the PREVENT-TB trial (85% vs 81%) [30]. However, better treatment tolerability of levofloxacin and hence completion rate were observed within TB-CHAMP compared to the Vietnam Quinolones for MDR-TB Trial (ACTRN12616000215426), which enrolled mainly adults with household MDR-TB exposure in Vietnam [8]. In the Vietnam Quinolones for MDR-TB Trial, 70% of participants taking levofloxacin versus 85% of those on placebo achieved adequate treatment, with the levofloxacin group more likely to discontinue treatment for adverse events or because of the participants’ own decision [7]. Furthermore, a combined analysis of the 2 trials found the likelihood of stopping levofloxacin TPT early for adverse events increased with age [7].

Our findings that baseline demographic and clinical characteristics did not strongly predict treatment discontinuation for nonclinical reasons are somewhat consistent with previous studies for drug-susceptible TPT [28, 31–33]. Treatment acceptability by the child and caregiver and behavioral factors were more important determinants. This highlights the challenge in identifying individuals at increased risk of TPT noncompletion from pretreatment characteristics alone, and the need to respond to early signs of poor acceptability and/or adherence. The likelihood of treatment noncompletion for nonclinical reasons and that of poor adherence varied between sites, being lowest in more rural areas, suggesting there may be other factors not accounted for.

We found that stopping treatment early for nonclinical reasons occurred more frequently in children with reported use of herbal/traditional medicine. This has also been observed for treatment of TB disease [26, 34]. Prevalence of herbal/traditional medicine use was higher at the 2 more rural sites in the study. Study staff reported local communities in the North-West and Northern Cape provinces traditionally make tea from certain plants often grown in gardens, which are taken preemptively in cold seasons to prevent colds and flu. When someone develops TB symptoms, as these are often similar to flu symptoms, family members may drink this tea to prevent illness. In addition, potential preference for traditional treatments among caregivers could be due to cultural beliefs or concerns about potential side effects of TPT medication [12, 15].

Treatment acceptability at the baseline visit after the first dose was administered was predictive of how well children completed treatment subsequently. Children who had to be coerced to take the medication, and those whose caregivers found administering the first dose difficult, were more likely to discontinue treatment early for nonclinical reasons. Acceptability at the week 4 visit remained predictive of subsequent treatment retention, although these data were sparse, partly due to a general improvement in acceptability over time in the overall study cohort [21].

Levofloxacin has an extremely bitter taste that is difficult to mask when crushed or softened. Size of pills and bitter taste are well-known key barriers to TPT adherence in children [12, 18]. Separate analyses within TB-CHAMP previously found the adult levofloxacin formulation was more difficult to prepare and administer than the placebo, and was disliked by many younger children [17, 21]. Moreover, participants who could not swallow the tablets halved or whole reported poorer acceptability. This may explain the weak association we observed between younger age and early treatment discontinuation for nonclinical reasons. Improving access to the better-tasting dispersible levofloxacin formulation is a priority for enhancing acceptability and treatment completion in young children, particularly as they have the highest risk of developing TB after exposure [3, 17].

We found early behavioral predictors of treatment discontinuation for nonclinical reasons included poor adherence while on treatment, not bringing in pill bottles, and not returning treatment cards by week 4. This concurs with findings from postanalyses of trials comparing 4 months of rifampicin to 9 months of isoniazid for TPT in adults, which additionally showed the likelihood of premature treatment discontinuation increased markedly as the number of behavioral predictors of discontinuation increased [28, 35]. These behavioral indicators, easily identifiable during treatment follow-up, are potentially useful triggers for appropriate interventions to improve adherence during routine care.

The strengths of this analysis include the large cohort size of more than 900 participants, with good follow-up and completeness of data on treatment adherence. To facilitate interpretation and better understanding of the barriers to treatment completion, we accounted for treatment being stopped early for nonclinical reasons versus for clinical reasons. The inclusion of data on acceptability and behavioral factors, alongside sociodemographic and clinical data, enabled a comprehensive assessment of the predictors of treatment noncompletion.

Several considerations are worth noting. The analysis included mainly young children younger than age 5 years; findings may not be generalizable to older children and adolescents. Residual confounding may account for some of the associations observed. Use of herbal/traditional medicine was likely to be underreported in older children because of recall bias, though the association between herbal/traditional use and early treatment discontinuation for nonclinical reasons remained when analysis was restricted to children younger than 2 years of age. The excellent adherence observed while children were on treatment may partly be due to “survivor” bias whereby those with poor adherence could be more likely to have stopped treatment early. Finally, treatment adherence and completion may be poorer in programmatic settings due to issues such as health system infrastructure (eg, staff resources), knowledge gaps among healthcare workers and caregivers, and access to care [12, 36]. A systematic review found over half of studies of child contact management under programmatic settings in high TB-burden countries reported completion rates of below 50% for drug-susceptible TPT mainly with isoniazid monotherapy [12].

With the roll-out of MDR-TPT in high TB-burden settings, optimizing treatment adherence and completion in children is an important aspect of strengthening the contact management cascade of care. Although treatment completion with the levofloxacin adult formulation was good overall among children, counseling and support for caregivers are important, as is addressing early signs of poor adherence. Access to child-friendly formulation of levofloxacin for young children, along with development of new shorter MDR-TPT regimens, remain essential [36].

## Supplementary Material

ofaf425_Supplementary_Data

## Supplementary Data

Supplementary materials are available at *Open Forum Infectious Diseases*  online. Consisting of data provided by the authors to benefit the reader, the posted materials are not copyedited and are the sole responsibility of the authors, so questions or comments should be addressed to the corresponding author.

## Notes

***Author Contributions***. T.D. conceptualized the analyses with input from A.C.H., J.A.S., H.S.S., and S.E.P. J.B. performed the analysis, with support from T.D. T.D. wrote the paper with input from A.C.H., J.A.S., H.S.S., S.E.P., and J.B. All authors reviewed the submitted and final version of the manuscript and approved it.

***Acknowledgments***. The authors acknowledge the TB-CHAMP teams at all 4 sites and all participants and their caregivers. We also thank our community advisory boards and local TB services.

***Disclaimer***. The content of the manuscript is solely the responsibility of the authors. The funders had no role in study design, data collection and analysis, decision to publish, or preparation of the manuscript.

***Data availability***. The dataset is available upon request.

***Financial support.*** The TB-CHAMP trial was supported by UNITAID, through the BENEFIT Kids project grant [grant number 2019-36-SUN-MDR] to Stellenbosch University. UNITAID accelerates access to innovative health products and lays the foundations for their scale-up by countries and partners. This trial was also funded by a Joint Global Health Trials (JGHT) grant to Stellenbosch University [grant number MR/M007340/1], supported by the Department of Health and Social Care (DHSC), the Foreign, Commonwealth & Development Office (FCDO), the Global Challenges Research Fund (GCRF), the Medical Research Council United Kingdom (MRC) and Wellcome. This UK-funded award is part of the EDCTP2 programme supported by the European Union. Additional funding was provided by the South African Medical Research Council for the TB-CHAMP trial grant to Stellenbosch University and the South African National Research Foundation to ACH (SARCHi chair). The Medical Research Council Clinical Trials Unit at University College London received core support from the Medical Research Council United Kingdom [grant numbers MC_UU_00004/04, MC_UU_00004/09]. The funders had no rule in the design, implementation and dissemination of these results. No funding was provided by Macleod's Pharmaceuticals. Study drug was procured for the trial.
