# Supplementary material for: Factors Predictive of Early Discontinuation of Preventive Treatment in Children With Household Exposure to Multidrug-resistant Tuberculosis
Source: Open Forum Infect Dis. 2025 Jul 18;12(8):ofaf425. doi: 10.1093/ofid/ofaf425 (PMC12378377; doi:10.1093/ofid/ofaf425)
Supplement: ofaf425_Supplementary_Data [file ofaf425_supplementary_data.docx]

**SUPPLEMENTARY APPENDIX**

**Factors predictive of early discontinuation of preventive treatment in children with household exposure to multidrug-resistant tuberculosis**

**
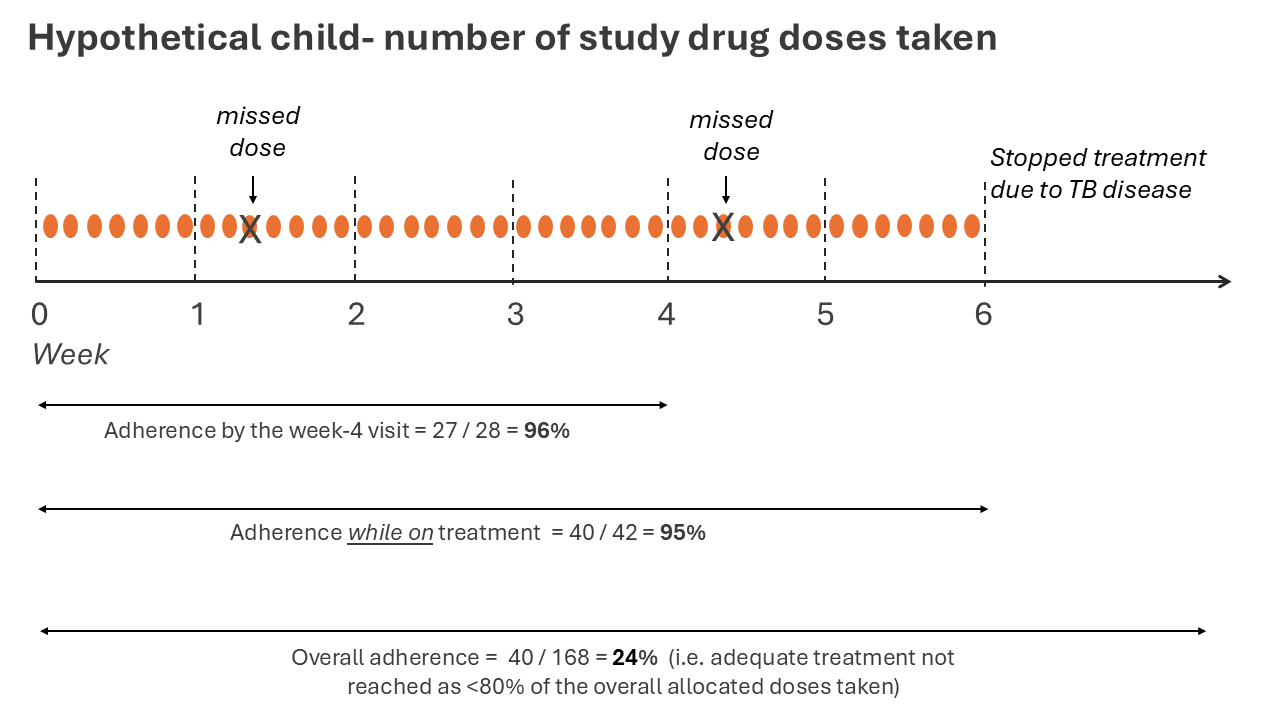
**

**Figure S1. Defining (i) adherence by the week-4 visit (ii) adherence while on treatment and (iii) adequate treatment**

**
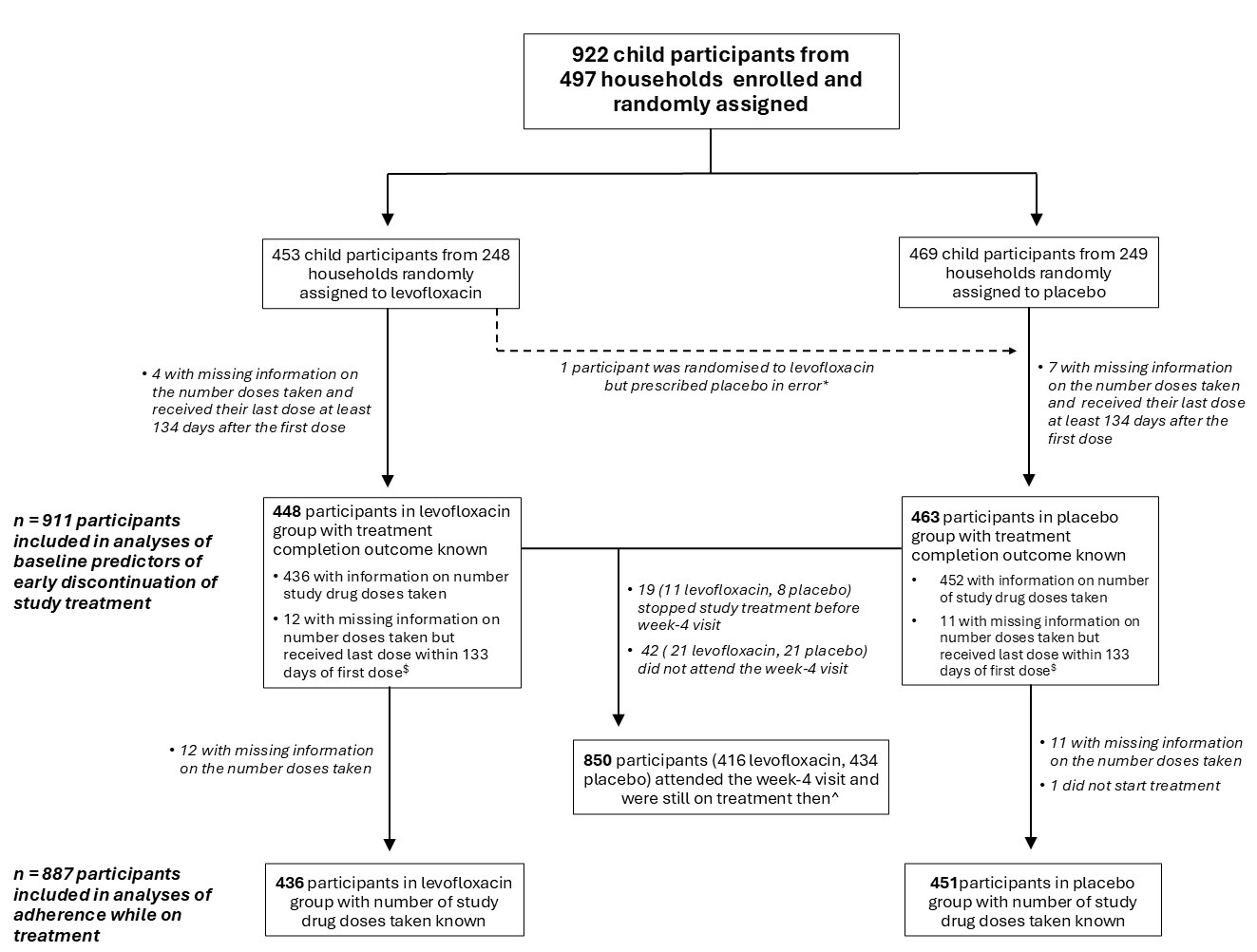
**

**Figure S2. Flow of participants included in analyses**

* Participant was analysed under placebo group for the purpose of these analyses.

$ Participants were assumed to have taken <80% of the overall 168 doses.

^ These were included in the analyses assessing factors at the week-4 visit associated with subsequent early discontinuation of study treatment for non-clinical reasons.

**Table S1. Dosing of levofloxacin / placebo (250 mg tablets) by pre-defined weight-bands within the TB-CHAMP trial**

| **Weight bands (kg)** | **Levofloxacin / Placebo**  **250mg tablets*** |
| --- | --- |
| 3 - 4.9 | 0.25 tablet |
| 5 - 6.9 | 0.5 tablet |
| 7 - 9.9 | 0.5 tablet |
| 10 - 11.9 | 1 tablet |
| 12 - 15.9 | 1 tablet |
| 16 - 19.9 | 1.5 tablets |
| 20 - 24.9 | 1.5 tablets |
| 25 - 29.9 | 2 tablets |
| 30 - 39.9 | 2.5 tablets |
| ≥ 40 | 3 tablets |

***** Dosing was based on 15-20 mg/kg (maximum 750mg).

**Table S2. Characteristics of participants at baseline - additional information (N=911)**

|  | | Levofloxacin | Placebo | Total |
| --- | --- | --- | --- | --- |
| Number of children | N | 448 (100%) | 463 (100%) | 911 (100%) |
| BCG vaccination | No | 27 (6%) | 25 (5%) | 52 (6%) |
|  | Yes | 419 (94%) | 436 (95%) | 855 (94%) |
|  | Missing data | 2 | 2 | 4 |
| Currently on TB preventative therapy | No | 439 (98%) | 457 (99%) | 896 (98%) |
|  | Yes | 9 (2%) | 6 (1%) | 15 (2%) |
| Any TB symptoms | No | 370 (83%) | 398 (86%) | 768 (84%) |
|  | Yes | 78 (17%) | 65 (14%) | 143 (16%) |
| Weight-for-age Z score* | Median (IQR) | -0.5 (-1.2, 0.3) | -0.4 (-1.2, 0.4) | -0.4 (-1.2, 0.3) |
|  | ≤ -1 | 134 (30%) | 143 (31%) | 277 (30%) |
|  | -1 to <0 | 164 (37%) | 151 (33%) | 315 (35%) |
|  | ≥ 0 | 150 (33%) | 169 (36%) | 319 (35%) |
| Height-for-age Z score* | Median (IQR) | -0.9 (-1.6, -0.2) | -0.9 (-1.8, -0.2) | -0.9 (-1.7, -0.2) |
|  | ≤ -1 | 212 (47%) | 219 (47%) | 431 (47%) |
|  | -1 to <0 | 148 (33%) | 142 (31%) | 290 (32%) |
|  | ≥ 0 | 88 (20%) | 102 (22%) | 190 (21%) |

| Received antibiotics in last 2 weeks | No | 384 (86%) | 389 (84%) | 773 (85%) |
| --- | --- | --- | --- | --- |
|  | Yes | 60 (14%) | 72 (16%) | 132 (15%) |
|  | Missing data | 4 | 2 | 6 |
| Hospitalization in the last 6 months | No | 428 (96%) | 441 (95%) | 869 (96%) |
|  | Yes | 18 (4%) | 22 (5%) | 40 (4%) |
|  | Missing data | 2 | 0 | 2 |
| On ART if HIV-positive | No | 2 (20%) | 3 (33%) | 5 (26%) |
|  | Yes | 8 (80%) | 6 (67%) | 14 (74%) |
| Hours spent per day with index patient | 0-4 hours | 71 (16%) | 76 (16%) | 147 (16%) |
|  | 5-8 hours | 115 (26%) | 121 (26%) | 236 (26%) |
|  | 9-12 hours | 78 (17%) | 81 (17%) | 159 (17%) |
|  | More than 12 hours | 184 (41%) | 185 (40%) | 369 (41%) |
| Weeks from index patient starting TB treatment to first household child contact enrolment | ≤12 weeks | 337 (79%) | 396 (88%) | 733 (84%) |
|  | >12 weeks | 87 (21%) | 52 (12%) | 139 (16%) |
|  | Missing data | 24 | 15 | 39 |

* Weight‑for‑age z scores for children 10 years of age or younger were calculated with the use of the World Health Organization (WHO) reference standard. Scores were calculated with the use of the U.K. reference standard for children older than 10 years of age, because WHO weight‑for‑age growth charts are only available for children 10 years of age and younger. Height‑for‑age z scores were calculated with the use of the WHO reference standard.

IQR=interquartile range.

**Table S3. Participants who have ever received herbal or traditional medicine by enrolment**

| Factors |  | Number of children ever received herbal or traditional medicine / Total (%) | |
| --- | --- | --- | --- |
| Overall |  |  |  |
| Age (years) | <3 | 69 / 478 | (14.4%) |
|  | 3 to <5 | 45 / 350 | (12.9%) |
|  | ≥5 | 4 / 82 | (4.9%)* |
| Site | DTTC | 33 / 450 | (7.3%) |
|  | Shandukani | 7 / 166 | (4.2%) |
|  | Matlosana^$^ | 74 / 255 | (29.0%) |
|  | ILTBRU^$^ | 2 / 7 | (28.6%) |
|  | THINK | 2 / 32 | (6.2%) |

* Likely under-reporting among older children.

$ Matlosana and ILTBRU were in more rural areas while other sites were in more urban areas.

DTTC= Desmond Tutu TB Centre; ILTBRU=Isanga Lethemba TB Research Unit; THINK= TB and HIV Investigative Network.

**Table S4. Reasons for taking herbal or traditional medication (n=118)**

| Reasons | Number of children |
| --- | --- |
| traditional/religious/cultural | 9 |
| improve general well-being | 3 |
| prophylaxis* | 32 |
| protection/strengthening | 6 |
| abdominal | 9 |
| chest infection | 8 |
| constipation | 4 |
| diarrhoeal | 4 |
| fever | 2 |
| not growing well | 3 |
| skin rash | 4 |
| trauma/burns | 1 |
| wind | 21 |
| other | 12 |

* Reported by participants at the Matlosana site only.

**Table S5. Early discontinuation of treatment before reaching 80% allocated doses**

|  | Levofloxacin  (n=448) | Placebo  (n=463) | Overall  (n=911) |
| --- | --- | --- | --- |
| **1. Discontinued treatment early for clinical reasons before reaching 80% allocated doses** | 27 (6.0%) | 37 (8.0%) | 64 (7.0%) |
| Reason for stopping treatment |  |  |  |
| Late screening failures | 13 | 12 | 25* |
| Presumed/diagnosed TB disease | 1 | 11 | 12 |
| Died | 0 | 1 | 1 |
| Adverse event | 6 | 0 | 6 |
| New exposure to new drug-susceptible TB | 7 | 12 | 19 |
| Other | 0 | 1 | 1 |
| Cumulative incidence (95% CI) |  |  |  |
| By 12 weeks | 4.4% (2.8% - 6.7%) | 6.5% (4.5%-9.0%) | 5.5% (4.1%-7.1%) |
| By 24 weeks | 6.0% (4.1%-8.5%) | 8.0% (5.8%-10.7%) | 7.0% (5.5%-8.8%) |
| Sub-hazard ratio (95% CI) of treatment discontinuation for clinical reason, levofloxacin vs. placebo | 0.73 (0.38 -1.43), P=0.36 | |  |
|  |  |  |  |
| **2. Discontinued treatment early for non-clinical reasons before reaching 80% allocated doses** | 37 (8.3%) | 34 (7.3%) | 71 (7.8%) |
| Reasons for stopping treatment |  |  |  |
| Moved away | 10 | 13 | 23 |
| Withdrew consent | 17 | 15 | 32 |
| Lost to follow-up | 3 | 5 | 8 |
| Non-adherent | 5 | 1 | 6 |
| Other | 2 | 0 | 2 |
| Cumulative incidence (95% CI) |  |  |  |
| By 12 weeks | 5.8% (3.9%-8.2%) | 5.6% (3.8%-8.0%) | 5.7% (4.3%-7.3%) |
| By 28 weeks | 8.3% (5.9%-11.0%) | 7.3% (5.2%-10.0%) | 7.8% (6.2%-9.7%) |
| Sub-hazard ratio (95% CI) of treatment discontinuation for non-clinical reasons, levofloxacin vs. placebo | 1.12 (0.64 - 1.94), P=0.69 | |  |

* Reasons for late screening failure were: TB disease at baseline (n=6), delay in isoniazid sensitivity result for the index patient (n=14), index patient with fluoroquinolone-resistant multi-drug resistant TB (n=4), and other (n=1).

CI= confidence interval


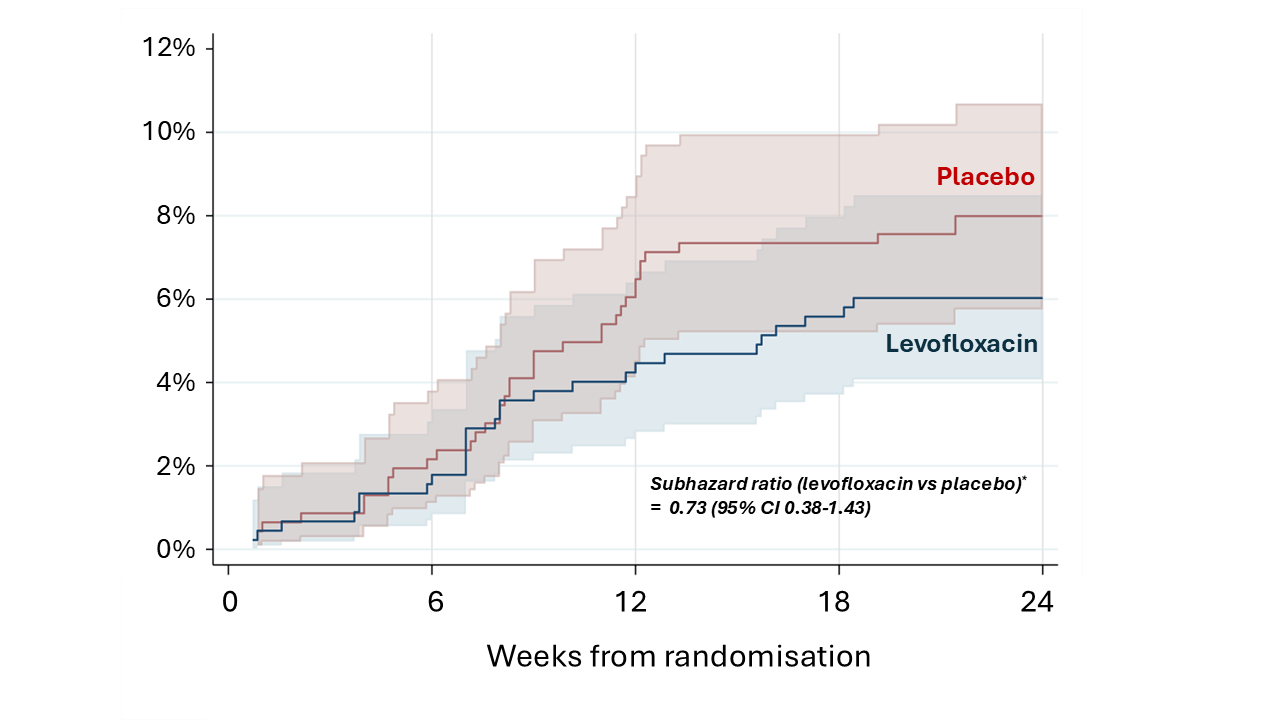


**Figure S3. Cumulative incidence of early discontinuation of treatment for clinical reason(s) before achieving adequate treatment (with 95% confidence bands)**

* The subhazard ratio was derived from Fine-Gray model.

CI= confidence interval

| **Treatment group** | **Number of children** | **% of doses taken based on that prescribed while on treatment** | | | **Odds ratio***  **(95% CI)** |
| --- | --- | --- | --- | --- | --- |
|  |  | **≥90%** | **80-89.9%** | **<80%** |  |
| Placebo | 451 | 398 (88%) | 41 (9%) | 12 (3%) | 1 |
| Levofloxacin | 436 | 359 (82%) | 52 (12%) | 25 (6%) | 1.60 (1.04,2.45)  P=0.031 |

**Table S6. Adherence while on treatment by study treatment group (N=887)**

* Estimated using ordinal logistic regression. An odds ratio greater than 1 corresponds to increased likelihood of poorer adherence.

CI= confidence interval

**Table S7. Multivariable analyses of factors associated with adherence while on treatment* (N=887^$^)**

| **Factors^** | | **Number of children** | **% of doses taken based on that prescribed while on treatment** | | | **Odds ratio^+^**  **(95% confidence interval)** | **P** |
| --- | --- | --- | --- | --- | --- | --- | --- |
|  | |  | **≥90%** | **80-89.9%** | **<80%** |  |  |
| Age (years) | <3yrs | 460 | 400 (87%) | 44 (10%) | 16 (3%) | 1 | 0.201 |
|  | 3 to <5 | 345 | 286 (83%) | 41 (12%) | 18 (5%) | 1.43 (0.96,2.15) |  |
|  | ≥5yrs | 82 | 71 (87%) | 8 (10%) | 3 (4%) | 1.01 (0.45,2.26) |  |
| Coughing history of index patient | No current cough | 436 | 374 (86%) | 44 (10%) | 18 (4%) | 1 | 0.143 |
|  | Current cough <4 weeks | 203 | 183 (90%) | 15 (7%) | 5 (2%) | 0.84 (0.46,1.53) |  |
|  | Current cough ≥4 weeks | 242 | 196 (81%) | 34 (14%) | 12 (5%) | 1.47 (0.92,2.38) |  |
| Height-for-age Z score | -2 to <-1 | 421 | 355 (84%) | 51 (12%) | 15 (4%) | 1 | 0.131 |
|  | -1 to <0 | 282 | 249 (88%) | 22 (8%) | 11 (4%) | 0.65 (0.42,1.00) |  |
|  | ≥0 | 184 | 153 (83%) | 20 (11%) | 11 (6%) | 0.97 (0.59,1.58) |  |
| Site^&^ | DTTC | 435 | 354 (81%) | 56 (13%) | 25 (6%) | 1 | <0.001 |
|  | Shandukani | 163 | 134 (82%) | 20 (12%) | 9 (6%) | 0.91 (0.53,1.59) |  |
|  | Matlosana | 250 | 232 (93%) | 16 (6%) | 2 (1%) | 0.32 (0.18,0.59) |  |
|  | THINK | 32 | 30 (94%) | 1 (3%) | 1 (3%) | 0.29 (0.06,1.37) |  |

* See Supplement Figure S1 for definition of adherence while on treatment.

$ Analyses excluded 24/911 children; 1 child did not start study treatment, and 23 children were missing information on the number of study drug doses taken.

^ Factors with P<0.2 in multivariable analysis and *a* *priori* confounders (age group and site) are presented.

+ Estimated using ordinal logistic regression. An odds ratio greater than 1 corresponds to increased likelihood of poorer adherence.

& The analysis comparing sites excluded one site (ILTBRU) with 7 children.
